# Editorial: Ectopic Mineralization of Tissues: Mechanisms, Risk Factors, Diseases, and Prevention

**DOI:** 10.3389/fcell.2021.759702

**Published:** 2021-10-25

**Authors:** Hervé Kempf, Svetlana Komarova, Monzur Murshed

**Affiliations:** ^1^UMR 7365 Université de Lorraine, CNRS, IMoPA, Nancy, France; ^2^Faculty of Dentistry, McGill University, Montreal, QC, Canada; ^3^Shriners Hospital for Children, Montreal, QC, Canada; ^4^Department of Medicine, McGill University, Montreal, QC, Canada

**Keywords:** calcification, pathologic, chronic disease, rare disease, mouse model

Extracellular matrix mineralization is an essential physiologic process in the skeleto-dental tissues. On the other hand, “soft” tissue mineralization (ectopic calcification), associated with several genetic and chronic metabolic diseases may result in serious health complications. Although these two processes are governed by multiple common determinants and overlapping mechanisms, all “soft” tissue calcification events cannot be categorized as cases of ectopic bone formation. The current special Research Topic presents a collection of original research and review articles covering various types of ectopic calcification events in multiple “soft” tissues (Figure 1).

**Figure 1:**
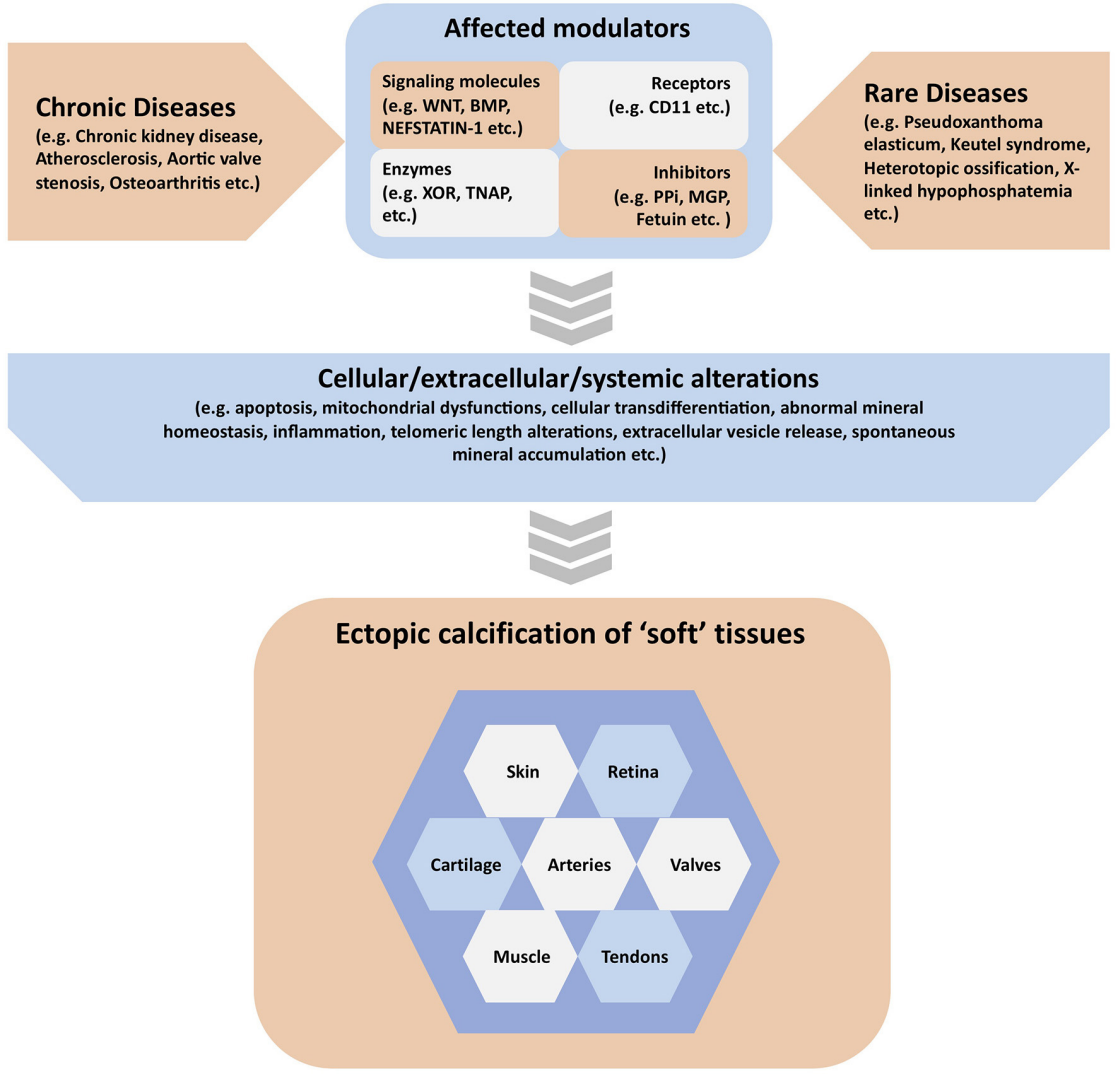
Graphical summary of the contents covered in the manuscripts published in the special issue. Ectopic calcification in various “soft” tissues can be associated with both chronic and rare diseases. These diseases may affect one or more modulators of “soft” tissue calcification, which include but are not limited to signaling molecules, receptors, enzymes, and other proteins or inorganic molecules acting as mineralization inhibitors. These activating or inhibiting modulators may in turn alter a variety of cellular, extracellular and systemic parameters in the extracellular matrix causing the initiation and progression of ectopic calcification in various “soft” tissues.

## Ectopic Mineralization Is a Latent and Tightly Regulated Process in “Soft” Tissues

Physiological and pathological mineralization most often results from the deposit of phosphocalcic crystals in the final form of hydroxyapatite within the extracellular matrix (ECM) deposited by the cells. Complex mechanisms are at work in the process leading to the ECM trapping of calcium phosphate salts, whose transition from amorphous to stable forms are governed by elaborate laws of thermodynamics as examined by Millán et al. in this special issue. In normal conditions however, precipitation of crystals of calcium and inorganic phosphate (P_i_) in “soft” tissues is actively prevented by various inhibitors including inorganic pyrophosphate (PP_i_; a dimer of P_i_). The PPi levels are mostly regulated by Tissue non-specific alkaline phosphatase/TNAP, which cleaves PP_i_ into two molecules of P_i_. As a result, TNAP plays dual functions as a remover of a mineralization inhibitor (PP_i_) and a generator of an inducer (P_i_) of mineralization, which make it an important regulator of physiologic mineralization in the “hard” tissues, but also potentially a key factor in pathological mineralization of “soft” tissues. Indeed, as described by Laurain et al.; Canet-Soulas et al.; Ehirchiou et al.; Nasi et al.; and Zhang et al., various pathologic conditions leading to the induction of TNAP expression or activity in the “soft” tissues may alter the crucial balance between PP_i_ and P_i_, thus favoring calcification. Additionally, Veiga-Lopez et al. also showed that PP_i_ levels controlled by TNAP links multiparity to cardiovascular risks in women.

Aside from the PP_i_ anion, a plethora of other inhibitors also prevent “soft” tissue calcification at local or systemic levels. For instance, Matrix Gla Protein (MGP) or fetuin-A are two major proteins that are involved in the regulation of ECM mineralization. MGP prevents ECM mineralization locally. While MGP's modes of action may involve binding to calcium ions and calcium crystals, as well as the inhibition of BMP signaling to inhibit osteogenesis in the “soft” tissues (Cancela et al.; Zhang et al.), the later mechanism needs to be confirmed in a direct genetic experiment. Unlike MGP, liver-derived plasma fetuin A primarily acts systemically by trapping calcium-phosphate mineral species into calciprotein particles (CPP) first as monomers (CPM) and then as larger structures, eventually cleared by the kidneys. The presence of this pathway *in vivo* has been elegantly demonstrated by Koeppert et al. in this special issue.

Besides inhibitors that hinder the development of ectopic calcification in soft tissues, numerous other signaling molecules/pathways have been implicated in the onset and development of ectopic calcification. In the current topic, papers highlighted the roles of Wnt signaling pathway in aortic valve stenosis (Khan et al.), nefstatin-1 in heterotopic ossification (HO) (Xu et al.), and xanthine oxidoreductase (Nasi et al.), CD11b (Ehirchiou et al.) or various forms of calcium crystals (Meyer et al.) in osteoarthritis (OA).

## Cellular Processes Associated With Ectopic Mineralization

At the cellular level, various processes are associated with the appearance and development of ectopic calcification. In the current topic a few of them were highlighted, including the formation of extracellular vesicles (Mansour et al.; Canet-Soulas et al.), inflammation (Koeppert et al.; Artiach and Bäck), apoptosis (Canet-Soulas et al.; Koeppert et al.), and mitochondrial dysfunction (Phadwal et al.; Lofaro et al.). Interestingly, Saraieva et al. identified telomere length as a novel potential contributor associated with valve calcification during aortic stenosis, although whether its involvement is a cause or consequence of the disease still needs to be clarified.

The trans-differentiation or switching of “soft” tissue cell types into mineralizing bone cells (chondrocytes or osteoblasts) (Mansour et al.; Cancela et al.) as a major mechanism of ectopic calcification has received a wide attention in the topic. A large variety of “soft” tissue cells, including cardiovascular cells, like vascular smooth muscle cells (Canet-Soulas et al.), valvular cells (Saraieva et al.; Khan et al.) and endothelial cells (Zhang et al.; Vasuri et al.) may undergo chondrogenic/osteogenic transdifferentiation. Two reviews by Canet-Soulas et al. and Zhang et al. provided outstanding overviews of the contributions of two types of vascular cells—the endothelial and vascular smooth muscle cells—in the vascular calcification process. Another review (Artiach and Bäck) and two experimental reports (Khan et al.; Saraieva et al.) highlighted the calcification events involving valvular cells during aortic valve calcification. Besides the intensely studied cardiovascular cells, gingival (Simancas et al.) or dermal (Lofaro et al.) fibroblasts, as well as tendocytes (Cauliez et al.) can also change their respective phenotype to calcifying cells. Slightly different cellular mechanisms take place for articular cartilage as in this particular tissue, the chondrocytes do not transdifferentiate, but instead terminally differentiate into hypertrophic chondrocytes, which actively participates in cartilaginous ECM calcification (Nasi et al.; Ehirchiou et al.; Meyer et al.). Additionally, an emerging concept in the field suggests that ectopic calcification can also occur without any involvement of chondrogenic/osteogenic differentiation of the cells present in the “soft” tissues (Canet-Soulas et al.; Cancela et al.).

## Ectopic Mineralization Can Occur During Chronic and Rare Diseases

Herein, reports showed the association of ectopic calcification with some chronic diseases, like chronic kidney disease (Laurain et al.; Millán et al.), atherosclerosis (Canet-Soulas et al.) or aortic valve stenosis (Artiach and Bäck; Khan et al.; Saraieva et al.). Three other reports explored the mechanisms of ectopic calcification in articular cartilage in patients with OA (Nasi et al.; Ehirchiou et al.) and chondrocalcinosis (Meyer et al.).

Heterotopic ossification (HO) is a significant health problem, which results in ectopic bone formation in the “soft” tissues, which are not the normal sites of osteogenesis. While the precise mechanism is not always well-understood, HO can occur as a rare disease due to genetic mutations, but it can also be acquired due to musculoskeletal trauma or central nervous system/nerve injury. In the current issue, Towler et al. characterized the great toe malformation in 41 patients suffering from fibrodysplasia ossificans progressiva (FOP), a rare genetic disease with constitutively active BMP signaling pathway and ectopic bone formation. Considering the benign nature of this trait in FOP patients, it has been ignored until now. Botman et al. examined the microarchitecture of the ectopically formed matured bones in two HO patients using non-invasive peripheral computed tomography scans. Interestingly, they found that the microstructures of the ectopic bones were more similar to that of the peripheral bones of a reference group than the FOP bones. These findings are important as only a limited number of studies reported such observations before. The article by Xu et al. examined the role on nesfatin-1, an adipokine, in osteogenic differentiation of tendon-derived stem cells and HO-associated pathologies in the tendons of a rat model. This study suggests that nestatin-1 adversely affects the tendon pathology in HO via the mTOR pathway. Finally, the review article by Girard et al. summarized the current understanding of the pathologies and mechanisms of neurogenic HO, a type of acquired HO.

The current topic includes two research articles on enthesopathies in a murine model (*Hyp*) of a rare disease called X-linked hypophosphatemia (XLH). While the major clinical feature in XLH is severe bone and tooth mineralization defects, primarily caused by phosphate wasting, multiple recent works show phosphate-independent pathologies in these patients and in the mouse model. Interestingly, both the patients and *Hyp* mice develop enthesopathies, paradoxically hallmarked by ectopic mineralization of enthesis fibrocartilage and articular cartilage. Faraji-Bellée et al. characterized the progressive joint anomalies of *Hyp* mice, suggesting the use of this model in future pre-clinical preventive studies. In a separate article, Cauliez et al. showed that the conventional treatment (phosphate supplement with calcitriol) does not improve the joint anomalies observed in *Hyp* mice.

Apart from HO and XLH, “soft” tissues calcification in several other rare genetic disorders or syndromes have been studied/reviewed in this special issue. Cancela et al. reviewed 50 years of research since the discovery of the Keutel syndrome in 1971. Keutel syndrome is caused by the loss-of-function mutations in MGP and the study highlights the significant findings involving human patients and cell and animal models. Another “classic” rare disease where multiple “soft” tissues including eyes, cardiovascular tissues or skin are calcified is pseudoxanthoma elasticum (PXE), caused by mutations in *ABCC6* or *ENPP1* gene. Lofaro et al. examined the dermal fibroblasts from human patients to examine the role of mitochondrial metabolism and how this may affect the extracellular milieu of the affected “soft” tissues. Although, fibroblasts do not express the *ABCC6* gene, the study was justified on the premise that ECM is a critical component to regulate mineral deposition in a tissue, and fibroblasts are among the major cell types synthesizing the ECMs in the affected tissues. If eventually proven, such a role for fibroblasts in the regulation of calcification may apply to other rare diseases. Lastly, an interesting study by Simancas Escorcia et al. describes how gingival fibroblasts transdifferentiate into chondrocytes/osteoblasts and eventually mineralize the surrounding ECM in the enamel renal syndrome caused by the complete loss of the pseudokinase FAM20A.

It is noteworthy that each of the rare diseases presented in this issue carries gain- or loss-of-function mutations in a specific gene (e.g., *ENPP1, ALK2, PHEX, ABCC6, FAM20A*, and *MGP*). For some of these genes, the effect of the mutations on the pathology is well-understood, while for some, it is still unknown. Considering the diverse functional properties and the differential cellular/extracellular localization of the proteins coded by these genes, it is likely that they involve multiple distinct mechanisms to regulate “soft” tissue calcification. However, it is also possible that at least some of these proteins may cross-talk and share overlapping pathways to regulate ECM mineralization. Moreover, phenotypic variabilities observed in these rare diseases could be due to modifier genes as reported by De Vilder et al. in PXE patients.

## Novel Advancements, Biomarkers and Potential Treatments for Ectopic Mineralization

The report by Peeters et al. is highly significant as it introduces sex as a relatively novel angle to be considered while studying ectopic calcification. It will be important to know how sex may affect the initiation and progression of ectopic calcification and the type of minerals deposited in various types of “soft” tissue calcification events. A thorough understanding of these events may lead to sex-dependent personalized treatment strategies for ectopic calcification.

With the increasing knowledge on the mechanisms of ectopic calcification, the field is facing two critical challenges: finding useful biomarkers for early detection of ectopic calcification events, and effective treatments to block or reverse this pathologic process occurring in both chronic and rare diseases. The current issue includes reports highlighting some biomarkers either studied in human cohorts (Peeters et al.) or through experimental data (Phadwal et al.). Regarding the potential targets for possible therapeutic treatments, Artiach and Back also discuss the potential of omega-3 therapy, known for its anti-inflammatory benefits, in treating the inflammation in aortic valve stenosis. In addition to natural components, synthetic drugs could also be effectively used. For instance, Mansour et al. showed that a synthetic peptide (GFOGER) modifies protein content of released extracellular vesicles in calcified vascular tissues and osteogenic switching of cultured cells *in vitro*. However, these findings need to be validated in animal models and eventually in human patients. The use of preclinical models, such as *Hyp* mice (Cauliez et al.) or MGP-deficient mice (Cancela et al.) is therefore critical before confirming the therapeutic potentials of the molecules in humans.

## Concluding Remarks

The special issue carries a total of 28 submissions from some of the leading experts in the field working on different types of ectopic calcification events. Although the types of ectopic calcification events presented are quite diverse, some diseases such as generalized arterial calcification in infancy and calcification associated with diabetes were not included. Also, none of the articles provided a thorough update on the available or promising treatment strategies. It will be important to cover these topics in future compilations. Future work in the field needs to focus on (i) the development of novel biomarkers to predict the progression of various ectopic calcification events, (ii) the characterization of new animal models and human pathologies associated with ectopic calcification, (iii) the understanding of the yet unknown mechanisms of action of some of the critical regulators of ECM mineralization, and most importantly, (iv) the development of effective therapies for various types of “soft” tissue calcification.

## Author Contributions

HK and MM drafted the manuscript. All authors revised, edited, and approved the final version of the editorial.

## Funding

This work was supported by the European Joint Program on Rare Diseases (EJP RD) through the project PhysPath-KS (EJPRD2019-290 to MM and HK).

## Conflict of Interest

The authors declare that the research was conducted in the absence of any commercial or financial relationships that could be construed as a potential conflict of interest.

## Publisher's Note

All claims expressed in this article are solely those of the authors and do not necessarily represent those of their affiliated organizations, or those of the publisher, the editors and the reviewers. Any product that may be evaluated in this article, or claim that may be made by its manufacturer, is not guaranteed or endorsed by the publisher.

